# Decrease in the Level of Nervonic Acid and Increased Gamma Linolenic Acid in the Plasma of Women with Polycystic Ovary Syndrome after a Three-month Low-glycaemic Index and Caloric Reduction Diet

**DOI:** 10.1515/biol-2019-0026

**Published:** 2019-07-10

**Authors:** Małgorzata Szczuko, Arleta Drozd, Dominika Maciejewska, Marta Zapałowska-Chwyć, Ewa Stachowska

**Affiliations:** 1 Department of Biochemistry and Human Nutrition, Pomeranian Medical University in Szczecin, Szczecin Poland; 2 Clinic of Gynecology and Urogynecology, Pomeranian Medical University in Szczecin, Szczecin Poland

**Keywords:** polycystic ovary syndrome, fatty acids, nervonic acid, gamma linolenic acid, dietary intervention, reduction diet, glycaemic index

## Abstract

The aetiology of polycystic ovary syndrome (PCOS) remains uncertain and thus dedicated studies are still of much importance. Patients in this group are at high risk for metabolic syndrome, diabetes and ischemic heart disease. Our goal was to use a dietary intervention, facilitating the regression of the disease, through the observation of lipid and hormonal profiles, carbohydrate metabolic parameters and metabolomics of plasma fatty acids. There were 39 Caucasian women with PCOS aged 26.76 ±5.08 that qualified for this study. Fatty acid profiles were investigated using gas chromatography. The results of plasma fatty acids were compared with the initial results and the control group. A three-month caloric reduction diet with low glycemic index (GI) reduces the level of nervonic acid and is a great alternative in PCOS therapy. The introduction of rapeseed oil and olive oil to the lowered GI reduction diet caused the increase in the ratio of average length chain fatty acids (C10:0, C14:0) and the enhancement of synthesis pathways for pentadecanoic acid (C15:0) and gamma-linolenic acid (GLA, C18:3n-6), but did not inhibit the synthesis of the derivatives of arachidic acid (C20:0). Additionally, a decrease in the level of nervonic acid (C24:1) was observed. Biochemical analysis of blood showed the improvement of plasma lipid fractions, but a significant reduction of androgen levels was not observed.

A reduction diet with lowered GI lead to many positive effects in the improvement of the biochemical parameters of women with PCOS. It should be continued for a prolonged period of time, until the synthesis pathways for inflammatory factors are silenced.

## 1 Introduction

In an analysis of available literature devoted to optimal therapeutic diet strategies for women with PCOS (polycystic ovary syndrome), we noted that the best results were obtained by Marsh et al. using a reduction diet with low GI (glycaemicindex) and increased physical activity [1,2]. They showed that a low-GI reduction diet has a greater beneficial effect on menstrual cycle like more regular cycles (improvement in 95% of examined women) than a high-GI reduction diet (improvement in 63% of examined women). However, other diet modifications with respect to macronutrients, such as a high-protein diet, did not have a positive effect on the parameters studied by Moran et al. Moreover. A high-protein diet, due to the positive nitrogen balance, led to the increased synthesis of ammonia and urea, which overloaded the kidneys and liver [3,4]. A similar relationship is present during the consumption of a high-fat diet due to the formation of ketone bodies[5]. Ketogenic diets are usually rich in animal fats, which are more susceptible to the activity of reactive oxygen species and do not deliver appropriate amounts of antioxidants. This type of diet is also not a good solution for women with PCOS, as ketosis increases inflammation [6]. A different study showed that a low-calorie diet with an increased amount of polyunsaturated fatty acids (PUFA) resulted in decreased BMI but did not present significant changes in body composition and waist circumference [7]. Furthermore, Stamets et al. described an improvement in hirsutism in women with PCOS due to the increased amount of carbohydrates [8]. Therefore, a higher ratio of carbohydrates in a diet could be a good solution for some women with PCOS, but due to the consequences related to improper glycaemia, a high amount of dietary carbohydrates is unfavourable. Increasing evidence suggests that insulin resistance and secondary hyperinsulinemia together with hyperandrogenism play a key synergistic role in the development and maintenance of metabolic alterations and anovulation or irregular cycles in both obese and lean patients with PCOS. This is why treatment strategies aim at reducing insulin resistance and decreasing metabolic consequences. A low dose of insulin sensitizers and antiandrogens can normalise the levels of fetuin-A in the serum [9], whereas it has been demonstrated that Myo-Inositol (MI) and D-Chiro-Inositol (DCI) are clinically effective in the treatment of PCOS by improving the ovulation, quality of oocytes and pregnancy index [10-11].

Considering all the data above, we decided that caloric deficiency in a diet can be obtained from carbohydrates and animal protein and fats. We maintained a total ratio of fat at 30% because omega-3 fatty acids play a crucial role in the regulation of the immune system and the improvement of insulin susceptibility, cell differentiation and ovulation [12] by reducing the synthesis of prostaglandins [13]. However, excess n-6 PUFA (polyunsaturated fatty acids) can affect the first two of the three phases of tumour development, i.e., initiation and promotion [14]. We were cautious in using large amounts of PUFA (olive oil, rapeseed oil, and cod-liver oil to supplement eicosapentaenoic acid (EPA) and docosahexaenoic acid (DHA) and maintained a balance in the ratio of macronutrients in the diet with lowered GI. Cod-liver oil was a natural preparation from the Molers Company with the highest production quality. It was challenging to determine the time-scale for the diet, as in the available literature concerning diet therapy for PCOS, the diets lasted from 1 month to even 12 months, and the resulting effects varied (Stamets et al- 1 month, Palomba et al- 6 weeks, Moran et al- 16 weeks, Kasim Karakas et al- 3 months, Thomson et al- 20 weeks, and Marsh et al- 12 months) [1,3,7-8,15-16].

Our goals were to observe the effects of diet therapy based on rational nutrition and to determine the time necessary to perform the therapy leading to the improvement of the lipid and free fatty acid profile of the plasma. The primary objective was to silence the synthesis pathways of palmitic acid (C:16:0), arachidonic acid (C:20:4n) and nervonic acid (C:24:1), as these are the major cause of inflammation. Decreasing the inflammatory reaction will have a positive effect on attaining hormonal balance.

Fatty acids have a structural function in biological membranes as the compounds of phospholipids and glycolipids. Oleic and stearic acids play a crucial role in the developmental competence of oocytes [17]. Fatty acids such as nervonic acid (C:24:1) and dihomogamma-linolenic acid (C:20:3n6), a derivative of linoleic acid (C:18:2n6), have been proposed as biomarkers of PCOS [18]. Our study provides insight into the changes undergone in the metabolic processes of women with PCOS. Such knowledge on metabolomics in relation to the period of PCOS development can be of great importance for developing diagnostic and therapeutic strategies. Even if the changes undergone in metabolic processes in women with PCOS are not the only cause of the disease, we think that they may be highly important.

## 2 Materials and methods

### 2.1 Participants

Screening tests for the diagnosis of PCOS were performed for 39 women, aged 17 - 38 (26.76 ± 5.08), in the Clinic of Gynecology and Urogynecology, Pomeranian Medical University. The control group included potentially healthy women of similar age with appropriate body weight with respect to height. The diagnosis of PCOS was performed according to Rotterdam’s criteria using USG (Ultrasound Voluson 730, GE, Switzerland). The test group comprised Caucasian women aged 26.31 ± 5.52, with an average body weight of 80.98 ± 16.06 kg and an average height of 1.67 ± 0.06 m. The criteria for exclusion were as follows:

– hyperprolactinaemia, congenital adrenal hyperplasia, Cushing’s syndrome, acromegaly,
– pharmacotherapy: hormonal contraception, hypoglycaemic drugs such as metformin,
– pregnancy,
– age above 40,
– change in dietary habits directly before the study,
– lack of progress in body mass reduction due to not adhering to the recommendations.

**Figure 1:**
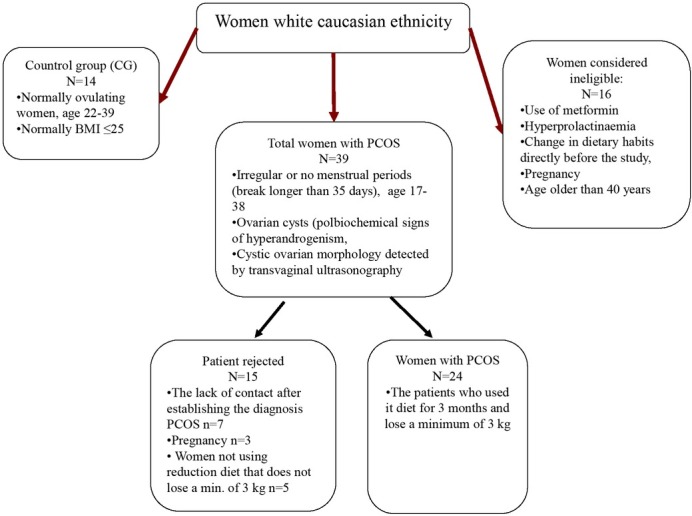
Research scheme

After three months of implementing the diet, 3 women were excluded from the test group due to pregnancy, 7 women did not appear for the control examination and 5 women did not follow the diet but only the recommendations and did not lose 3kg (in three months). Finally, the test group comprised 24 women with a PCOS CONSORT flow diagram of participant enrollment and biochemical analysis throughout the study by treatment group, where *n* is based on the total number of samples available for each visit (Figure 1).

All participants of the study were characterized by low physical activity, PAL index level = 1.3-1.4. One of the recommendations, provided by the diet, was to increase physical activity to at least 3 hours a week. The suggested forms of physical activity were nordic walking, swimming, aerobics, running, cycling, team games (such as volleyball, football, and handball), tennis, table tennis and dancing. The size of the trial for the population of 100000 was calculated by estimating the size of the fraction at the level of 10%. The confidence level was 95% and the acceptable error margin: 10%, which constituted 35 women.

**Informed consent** Informed consent has been obtained from all individuals included in this study

**Ethical approval** The research related to human use has been complied with all the relevant national regulations, institutional policies and in accordance the tenets of the Helsinki Declaration, and has been approved by the authors’ institutional review board.

### 2.2 Dietary intervention

Each woman received a seven-day menu and recommendations regarding the change of lifestyle, adjusted to individual caloric requirements. The compositions of the diets were calculated using the nutrition software Dieta 4.0, recommended by the National Food and Nutrition Institute. The diet caloricity was reduced by ca. 600 kcal. Each day included 5 meals: three main meals and 2 maintaining the level of glucose. All products on the menu were specified by weight. Food products used were the sources of all macronutrients, according to the food pyramid recommended by the National Food and Nutrition Institute in 2016. Five portions per day of the following products were used as the sources of carbohydrates: oatmeal, wholegrain rye bread or graham bread, brown rice, groats (wheat, millet and buckwheat), potatoes (sporadically), and/or wholemeal pasta. Selected carbohydrate products were characterized by a lowered glycaemic index.

Sources of protein (on average, 1 portion of meat and 2 portions of dairy products per day) in the recommended diet included the following products: eggs, lean meat without skin (turkey or chicken), fish (mainly sea fish such as sole, salmon, or tuna), semi-skimmed pasteurized milk and dairy products (quark, natural yoghurt and buttermilk with 2% fat), nuts and seeds (almonds, pumpkin seeds, sunflower seeds, sesame seeds and poppy seeds) and legumes (soy, red lentils, beans, and peas).

The sources of fat (on average, 2 portions per day) were raw oils (rapeseed oil or olive oil) and oily fruits such as avocado, as well as nuts, fish, meat and dairy products.

The diet also included fruits and vegetables with low GI. In agreement with the rules of rational nutrition, they were present in every meal, supplementing the diet with vitamins and minerals. Mushrooms were not included in the diet due to their difficult to digest

The following heat-treatment techniques were recommended to the patients to prepare the food: braising, roasting, cooking in water and steaming. Moreover, each patient was recommended to drink ca. 2 litres of fluid during the day. The recommended sources were iodized water and herbal infusions. The average weekly ratio of energy was up to 20% from protein, up to 30% from fat and up to 50% from carbohydrates. The consumption of cod-liver oil was recommended as a supplement to EPA (eicosapentaenoic acid) and DHA (docosahexaenoic acid). The contents of food nutrients, cholesterol and dietary fibre and the structure of macronutrients are presented in Table 1.

**Table 1:** Average contents of nutrients in 1600 and 1800 kcal diets.

Nutrient	Average	SD	Average	SD
Energy[kJ]	6930.79	437.18	7882.02	445.43
Energy [kcal]	1634.98	105.57	1822.16	107.53
Water [g]	1227.30	144.28	1321.30	151.28
Total protein [g]	84.61	10.99	85.52	12.41
Animal protein [g]	46.12	13.40	46.58	15.17
Plant protein [g]	35.85	8.64	37.30	9.36
Total fat [g]	54.05	8.54	62.64	9.43
Fatty acids:	14.57	2.86	18.07	4.63
Total saturated - total [g]				
Fatty acids: total monounsaturated [g]	23.47	5.21	26.28	4.30
Fatty acids: total polyunsaturated [g]	11.64	2.46	13.41	2.96
Cholesterol [g]	164.69	59.72	168.51	68.71
Total carbohydrates [g]	238.84	22.91	271.38	22.09
Digestible carbohydrates [g]	205.35	20.55	233.70	20.62
Ash [g]	15.41	0.46	17.26	0.65
Sodium [mg]	1402.94	375.14	1691.99	491.74
Potassium [mg]	4055.60	680.11	4421.33	649.54
Calcium [mg]	966.80	278.25	1032.31	279.11
Phosphorus [mg]	1728.82	167.04	1910.24	194.73
Magnesium [mg]	482.17	18.75	533.72	26.36
Iron [mg]	13.10	1.92	14.55	1.89
Zinc [mg]	12.19	0.86	13.70	1.08
Copper [mg]	1.66	0.27	1.84	0.25
Manganese [mg]	7.20	1.29	8.08	1.52
Vitamin A (Eq. retinol)	1725.46	765.24	1766.58	760.49
Retinol	187.17	60.07	220.02	64.56
Beta-carotene	9230.57	4348.32	9280.41	4331.15
Vitamin E	16.00	3.56	17.05	3.28
(Eq. alfa-tocopherol)				
Tiamin [mg]	1.65	0.24	1.81	0.22
Riboflavin [mg]	2.00	0.21	2.15	0.24
Niacin [mg]	21.31	2.38	24.04	1.83
Vitamin B6	2.66	0.60	2.89	0.58
Vitamin C [mg]	302.12	100.19	320.84	102.24
Cholesterol [g]	154.69	59.72	178.51	68.71
Sucrose [g]	17.14	5.34	20.73	6.29
Lactose [g]	17.19	9.48	18.31	8.76
Starch [g]	109.96	29.51	128.96	35.95
Dietary fibre [g]	33.49	5.41	37.68	4.88
Folacin/folic acid [μg]	557.26	122.99	593.52	117.26
Vitamin B12 [μg]	3.83	1.55	4.07	1.46
Vitamin D [μg]	3.47	3.77	3.52	3.76
Iodine [μg]	57.20	13.52	59.17	11.91
Percent of energy from protein [%]	20.77	2.73	20.16	2.79
Percent of energy from fat [%]	28.76	3.44	29.32	3.19
Percent of energy from carbohydrates [%]	50.47	4.63	50.52	4.38

### 2.3 Anthropometric measurements and blood biochemistry

All participants were subjected to anthropometric and bioelectrical impedance (BIA) measurements. The following measurements were taken: body mass (±0,1 kg), height (±0,5 cm), and circumference of the waist (±0,5 cm) and hip (±0,5 cm). BMI and WHR indexes were calculated from the obtained results. Using BIA 101 (Akern, Italy), we measured fat mass – FM (kg, %). After three months of following the diet, participants’ measurements were repeated.

Testosterone and insulin were assessed using ECLIA (electro-chemiluminescence immunoassay), androstenedione was determined using ELISA, the lipid profile was assessed with an enzymatic method with esterase and cholesterol oxidase, and glucose was determined with an enzymatic method with hexokinase.

### 2.4 Isolation of fatty acids

Plasma was obtained from whole-blood samples, which were collected in tubes containing EDTA as an anticoagulant. Plasma was extracted by centrifugation for 10 min at 1200 G and then stored at −80 °C until GC-MS analyses. The Folch method was used to extract the fatty acids for chromatographic analyses. In brief, 0.5 ml of plasma was saponified with 1 ml of 2mol/L KOH methanolic solution at 70 °C for 20 min and then methylated with 2 ml of 14% boron trifluoride in methanol under the same conditions. Next, 2 ml of n-hexane and 10 ml of saturated NaCl solution were added. For the analyses, we collected 1 ml of the n-hexane phase [19].

### 2.5 Analysis of fatty acid methyl esters

Gas chromatography was performed using the Agilent Technologies 7890A GC System (SUPELCOWAX™ 10) using a Capillary GC Column (15 mm × 0.10 mm, 0.10 μm; Supelco, Bellefonte, PA, United States). The following chromatographic conditions were used: initial temperature of 60°C for 0 min, increased at a rate of 40°C/min to 160°C (0 min), next increased at a rate of 30°C/min to 190°C (0.5 min) and then increased at a rate of 30°C / min to 230°C for 2.6 min, and then maintained for 4.9 min. The total analysis lasted ca. 8 min, and the gas flow rate was 0.8 ml/min. Hydrogen was used as the carrier gas. Fatty acids were identified by comparing their retention times with those of commercially available standards. C:18:2n6t, C:18:4 and C:20:3n3 cis-11 were not isolated in either the PCOS or control group.

### 2.6 Statistical analysis

Statistical analyses were performed with Statistica 12.0. The results are expressed as mean ± standard deviation. Because the distribution in most cases deviated from normal (Shapiro-Wilk test), we used a non-parametric Mann-Whitney test for comparisons between the groups, and *p*≤0.05 or p≤0.01 was considered statistically significant. To better visualise the changes in the proportions of particular fatty acids, the results were presented as the percentage ratio in the plasma, with 100% being the amount of fatty acids in the tested group. Significant differences in tested parameters between the groups were calculated using the analysis of variance (ANOVA) with post hoc Tukey’s tests for multiple comparisons, and p≤0.05 was considered statistically significant.

## 3 Results

### 3.1 Comparison of fatty acid contents before and after the diet (mg/dl; %)

When analysing the contents of fatty acids before and after the diet, we observed a statistically significant improvement in the group of women with PCOS with respect to 10 fatty acids (Table 2). The level of the average chain length fatty acids increased, including lauric acid (C:10:0), myristic acid (C14:0) and pentadecanoic acid (C15:0). We also noted an increased level of long-chain fatty acids, such as heptadecanoic acid (C:17:1 cis10), stearic acid (C:18:0), gamma-linolenic acid (C:18:3n-6), arachidic acid (C:20:0) and erucic acid (C:22:1n9). Additionally, the level of nervonic acid (C:24:1) had decreased significantly (Table 2).

**Table 2:** Quantification of fatty acids in plasma women with PCOS before and after the diet (mg/dl)

Fatty acids (mg/dl)	PCO_II** n=24		PCO_I* n=39		p value
		
	MEAN	SD	MEAN	SD	
**C8:0 caprylic acid**	0,363	1,258	0,499	0,422	NS
**C10:0 capric acid**	26,24	14,724	13,957	3,435	p<0,01
**C11:0 undecanoic acid**	0	0	0	0	NS
**C12:0 lauric acid**	5,013	10,715	0,660	0,526	NS
**C13:0 tridecanoic acid**	0	0	30,114	104,318	NS
**C14:0 myristic acid**	12,291	5,049	7,717	4,133	p<0,05
**C14:1 miristoleic acid**	0	0	0,139	0,358	NS
**C15:0 pentadecanoic acid**	4,535	1,024	1,223	0,605	p<0,01
**C15:1 cis-10-pentadecanoid acid**	0	0	0	0	NS
**C16:0 palmitic acid**	208,293	40,617	185,389	61,467	NS
**C16:1 palmitoleic acid**	14,754	5,978	14,715	9,011	NS
**C17:0 heptadecanoic acid**	2,651	0,938	2,052	0,561	NS
**C17:1 cis-10-heptadecanoid acid**	1,639	0,648	1,129	0,557	p<0,05
**C18:0 stearic acid**	110,336	14,787	65,129	11,567	p<0,01
**C18:1w9 oleic acid**	167,770	41,829	150,175	69,584	NS
**C18:1trans11 trans-vaccenic acid**	15,557	3,643	12,899	5,137	NS
**C18:2n6c linoleic acid**	179,008	26,393	176,170	42,684	NS
**C18:2n6t linoleic acid**	0	0	0	0	NS
**C18:3n-6 gamma-linolenic acid**	34,058	33,844	2,593	0,974	p<0,01
**C18:3n-3 linolenic acid**	5,527	2,538	5,272	2,591	NS
**C18:4 stearidonic acid**	0	0	0	0	NS
**C20:0 arachidic acid**	2,488	0,946	0,815	0,503	p<0,01
**C20:1 cis-11-eicosanoic acid**	1,681	1,105	1,634	1,000	NS
**C20:2 cis-11-eicodienic acid**	0	0	23,587	45,749	NS
**C20:3n6 cis-11-eicosatrienoic acid**	10,591	2,960	9,788	3,099	NS
**C20:4n6 arachidonic acid**	51,732	9,720	45,519	11,246	NS
**C20:3n3 cis-11-eicosatrienoic acid**	0	0	0	0	NS
**C20:5 EPA**	5,559	4,144	4,381	1,312	NS
**C22:0 behenic acid**	0,542	1,138	0,276	0,386	NS
**C22:1n9 erucic acid**	1,067	1,387	0	0	p<0,05
**C22:2 cis-docosadienoic acid**	0,121	0,420	0	0	NS
**C23:0 tricosanoic acid**	1,690	1,041	1,264	0,690	NS
**C22:4n6 docosatetraenic acid**	1,939	0,974	1,575	0,642	NS
**C22:5w3 docosapentaenoic acid**	2,919	1,474	3,299	0,964	NS
**C24:0 lignoceric acid**	0,314	1,089	0,036	0,123	NS
**C22:6w3 docosahexaenoic acid**	15,948	8,050	12,842	5,961	NS
**C24:1 nervonic acid**	1,289	4,069	20,781	12,101	p<0,01
**SFA**	248,340	60,465	278,791	120,273	NS
**MUFA**	520,501	77,636	244,439	110,878	p<0,01
**PUFA**	116,709	40,503	578,692	410,562	p<0,01
**Total UFA**	885,550	151,044	1263,926	744,201	p<0,01
**SFA/UFA**	0,279	0,037	0,270	0,124	NS

*PCO I-women with PCOS befor dietary intervension **PCO II- women with PCOS after dietary intervension

The analyses of the percentage ratio of fatty acids before and after the diet in the group of women with PCOS showed that the observed differences corresponded to the same fatty acids and correlations (Table 3).

**Table 3:** Quantification of fatty acids in plasma women with PCOS before and after the diet (%)

Fatty acids (%)	PCO_II** n=24		PCO_I* n=39		p value
		
	MEAN	SD	MEAN	SD	
**C8:0 caprylic acid**	0,038	0,153	0,062	0,041	NS
**C10:0 capric acid**	3,023	1,432	1,824	0,578	p<0,05
**C11:0 undecanoic acid**	0	0	0	0	NS
**C12:0 lauric acid**	0,548	1,316	0,0870	0,071	NS
**C13:0 tridecanoic acid**	0	0	2,998	10,007	NS
**C14:0 myristic acid**	1,456	0,660	0,931	0,293	p<0,05
**C14:1 miristoleic acid**	0	0	0,013	0,029	NS
**C15:0 pentadecanoic acid**	0,536	0,175	0,157	0,057	p<0,01
**C15:1 cis-10-pentadecanoid acid**	0	0	0	0	NS
**C16:0 palmitic acid**	23,368	1,025	23,102	4,219	NS
**C16:1 palmitoleic acid**	1,620	0,454	1,727	0,607	NS
**C17:0 heptadecanoic acid**	0,281	0,120	0,258	0,056	NS
**C17:1 cis-10-heptadecanoid acid**	0,174	0,086	0,134	0,038	NS
**C18:0 stearic acid**	12,630	1,502	8,397	1,752	p<0,01
**C18:1w9 oleic acid**	18,910	2,263	18,191	4,500	NS
**C18:1trans11 trans-vaccenic acid**	1,790	0,344	1,570	0,307	NS
**C18:2n6c linoleic acid**	20,787	2,498	22,106	3,395	NS
**C18:2n6t linoleic acid**	0	0	0	0	NS
**C18:3n-6 gamma-linolenic acid**	3,373	2,946	0,322	0,089	p<0,01
**C18:3n-3 linolenic acid**	0,610	0,330	0,652	0,253	NS
**C18:4 stearidonic acid**	0	0	0	0	NS
**C20:0 arachidic acid**	0,261	0,137	0,112	0,069	p<0,01
**C20:1 cis-11-eicosanoic acid**	0,184	0,129	0,205	0,086	NS
**C20:2 cis-11-eicodienic acid**	0,012	0,048	2,340	3,157	p<0,05
**C20:3n6 cis-11-eicosatrienoic acid**	1,145	0,416	1,245	0,294	NS
**C20:4n6 arachidonic acid**	5,865	1,104	5,832	1,356	NS
**C20:3n3 cis-11-eicosatrienoic acid**	0	0	0	0	NS
**C20:5n3 EPA**	0,562	0,454	0,560	0,169	NS
**C22:0 behenic acid**	0,050	0,107	0,040	0,055	NS
**C22:1n9 erucic acid**	0,267	0,659	0	0	p<0,05
**C22:2 cis-docosadienoic acid**	0,012	0,046	0	0	NS
**C23:0 tricosanoic acid**	0,180	0,120	0,170	0,080	NS
**C22:4n6 docosatetraenic acid**	0,204	0,110	0,206	0,065	NS
**C22:5w3 docosapentaenoic acid**	0,308	0,170	0,421	0,109	NS
**C24:0 lignoceric acid**	0,030	0,120	0,003	0,012	NS
**C22:6w3 docosahexaenoic acid**	1,650	0,892	1,584	0,522	NS
**C24:1 nervonic acid**	0,124	0,449	4,747	7,522	p<0,05
**SFA**	28,036	3,531	38,297	9,518	p<0,01
**MUFA**	59,416	2,361	24,484	6,768	p<0,01
**PUFA**	12,510	3,879	35,700	4,608	p<0,01
**SFA/UFA**	0,280	0,035	0,389	0,096	p<0,01

*PCO I-women with PCOS befor dietary intervension **PCO II- women with PCOS after dietary intervension

### 3.2 Comparison of fatty acid ratio (%) after the diet with the control group

**Figure 2:**
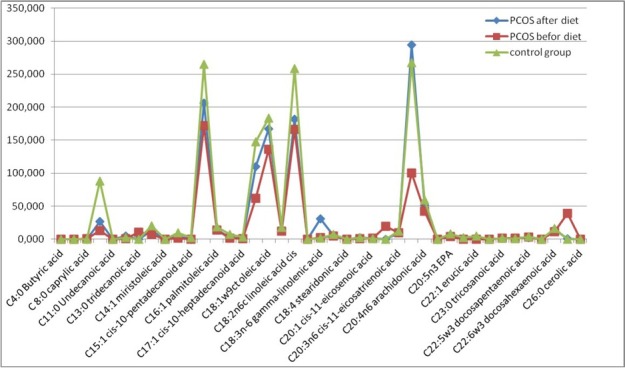
Changes in lipid profiles before and after the three-month dietary intervention in comparison to the control group

The analyses of the content of fatty acids in women with PCOS after dietary intervention, in comparison to the control group, still showed a statistically significantly lower content of average chain length fatty acids – saturated lauric acid (C:10:0) and monounsaturated pentadecanoic acid (C:15:1cis10) in women with PCOS. A statistically significantly lower ratio was also noted in the case of some long-chain fatty acids: heptadecanoic acid (C:17:0) and tricosanoic acid (C:23:4) (Table 4). However, in general, the long-chain fatty acids, and especially the derivatives of oleic acid, were elevated in women with PCOS in comparison to the control group. This was the case for oleic acid (C18:1w9), trans-vaccenic acid (C18:1trans11), gamma-linolenic acid (C:18:3n6), eicosanoic acid (C:20:1cis11) and arachidonic acid (C:20:4n6). A dramatic reduction in the content of nervonic acid (C:24:1) was observed. It was isolated in only one sample at the concentration several times lower than that before the diet. Changes in the lipid profile before and after dietary intervention, in comparison to the control group, are presented in Figure 2.

**Table 4:** Quantification of fatty acids between the group of plasma women with PCOS after dietary intervention and the control group (CG, %)

Fatty acids (%)	PCO_II** n=24		PCO_CG* n=14		p value
		
	MEAN	SD	MEAN	SD	
**C8:0 caprylic acid**	0,038	0,153	0	0	NS
**C10:0 capric acid**	3,023	1,432	9,253	4,735	p<0,05
**C11:0 undecanoic acid**	0	0	0	0	NS
**C12:0 lauric acid**	0,548	1,316	0,308	0,192	NS
**C13:0 tridecanoic acid**	0	0	0	0	NS
**C14:0 myristic acid**	1,456	0,660	1,745	0,325	NS
**C14:1 miristoleic acid**	0	0	0	0	NS
**C15:0 pentadecanoic acid**	0,536	0,175	0,550	0,097	NS
**C15:1 cis-10-pentadecanoid acid**	0	0	0,066	0,067	p<0,05
**C16:0 palmitic acid**	23,368	1,025	23,885	1,791	NS
**C16:1 palmitoleic acid**	1,620	0,454	1,474	0,453	NS
**C17:0 heptadecanoic acid**	0,281	0,120	0,508	0,178	p<0,05
**C17:1 cis-10-heptadecanoid acid**	0,174	0,086	0,133	0,098	NS
**C18:0 stearic acid**	12,630	1,502	13,551	1,353	NS
**C18:1w9 oleic acid**	18,91	2,263	15,145	2,084	p<0,01
**C18:1trans11 trans-vaccenic acid**	1,790	0,344	1,415	0,206	p<0,01
**C18:2n6c linoleic acid**	20,787	2,500	21,359	1,859	NS
**C18:2n6t linoleic acid**	0	0	0	0	NS
**C18:3n-6 gamma-linolenic acid**	3,373	2,946	0,243	0,179	p<0,01
**C18:3n-3 linolenic acid**	0,610	0,330	0,635	0,181	NS
**C18:4 stearidonic acid**	0	0	0	0	NS
**C20:0 arachidic acid**	0,261	0,137	0,307	0,198	NS
**C20:1 cis-11-eicosanoic acid**	0,183	0,129	0,072	0,106	p<0,05
**C20:2 cis-11-eicodienic acid**	0,012	0,048	0	0	NS
**C20:3n6 cis-11-eicosatrienoic acid**	1,145	0,416	1,070	0,274	NS
**C20:4n6 arachidonic acid**	5,865	1,104	5,153	0,650	p<0,05
**C20:3n3 cis-11-eicosatrienoic acid**	0	0	0	0	NS
**C20:5 EPA**	0,562	0,454	0,607	0,433	NS
**C22:0 behenic acid**	0,051	0,107	0,366	0,264	p<0,05
**C22:1n9 erucic acid**	0,267	0,659	0,039	0,064	NS
**C22:2 cis-docosadienoic acid**	0,012	0,046	0	0	NS
**C23:0 tricosanoic acid**	0,180	0,120	0,235	0,124	p<0,05
**C22:4n6 docosatetraenic acid**	0,204	0,110	0,055	0,088	NS
**C22:5w3 docosapentaenoic acid**	0,308	0,170	0,282	0,181	NS
**C24:0 lignoceric acid**	0,030	0,120	0,032	0,091	NS
**C22:6w3 docosahexaenoic acid**	1,650	0,892	1,499	0,456	NS
**C24:1 nervonic acid**	0,124	0,449	0,013	0,048	NS
**SFA**	28,036	3,531	29,789	3,325	NS
**MUFA**	59,416	2,361	61,268	2,687	NS
**PUFA**	12,510	3,879	8,942	1,117	p<0,01
**SFA/UFA**	0,281	0,035	0,298	0,033	NS

*CG- control group **PCO II-women with PCOS befor dietary intervension

### 3.3 Changes in families of fatty acids (SFA, MUFA, and PUFA)

Considering the particular groups of fatty acids (SFA, MUFA, and PUFA), an increased level (mg/dl) and ratio (%) of MUFA was observed after using a diet rich in vegetable oils (Table 1). Reductions were observed in the content (mg/dl) and ratio (%) of SFA and PUFA and the total content of all fatty acids (Table 2-3). However, when comparing the results with the control group (CG), an increased ratio of PUFA was noted (Table 4).

### 3.4 Comparison of biochemical parameters of blood before and after dietary intervention

When analysing anthropometric parameters of women with PCOS, it was observed that all analysed characteristics were improved after the diet (Table 5). Such improvement was not observed for the analyses of blood biochemical parameters. There was a decrease in almost all of the tested biochemical parameters except for HDL. However, statistically significant changes were observed only in the case of the reduction in lipid fractions: triglycerides by ca. 30 units, LDL by ca. 25 units and total cholesterol by ca. 20 units (Table 5). After the three-month reduction diet, no statistically significant changes were observed in androgen levels, even though a decrease in testosterone was visible.

**Table 5:** Antropometric and biochemical data of patients with PCOS before and after dietary intervention

Characteristic	PCOS-I before dietary intervention	PCOS-II after dietary intervention	P value
**Body mass [kg]**	83.75 ± 15.4	77.82 ± 15.3	0.000
**WC [cm]**	102.52 ± 14.51	94.79 ± 16.01	0.000
**HC [cm]**	109.79 ± 9.36	104.96 ± 9.14	0.000
**FM [%]**	39.14 ± 8.64	35.85 ± 6.25	0.002
**FM [kg]**	33.03 ± 11.45	29.68 ± 10.87	0.000
**BMI [kg/m^2^]**	30.13 ± 6.60	28.09 ± 6.33	0.000
**WHR**	0.93 ± 0.071	0.90 ± 0.10	0.012
**Glucose [mg/dl]**	92.95 ± 10.03	89.59 ± 6.68	NS
**Insulin [uU/ml]**	13.97 ± 10.86	10.65 ± 4.00	NS
**Cholesterol [mg/dl]**	179.13 ± 29.51	159.29 ± 27.21	0.019
**LDL [mg/dl]**	114.98 ± 32.93	89.62 ± 28.34	0.007
**TG [mg/dl]**	105.92 ± 51.87	76.62 ± 32.2	0.014
**HDL [mg/dl]**	54.44 ± 17.31	58.94 ± 17.23	NS
**Androstendion [ng/m]**	4.02 ± 1.56	4.25 ± 1.5	NS
**Testosterone [ng/m]**	0.69 ± 0.18	0.52 ± 0.14	NS

## 4 Discussion

### 4.1 Comparison of plasma fatty acid profile before and after dietary intervention

It is estimated that PCOS affects some percentage of women in developed countries, and the number of cases is constantly increasing [20]. Thus, finding the best and the least invasive therapy seems to be crucial in dealing with further health consequences. The introduction of olive oil and rapeseed oil to a diet, both oils rich in oleic acid with a comparatively low content of n-6 fatty acids, decreased the ratio of n-6 to n-3 fatty acids [21]. These vegetable oils are oxidatively stable and contain oleic acid, which limits the synthesis of liver cholesterol and triglycerides and thus intensifies esterification and cholesterol metabolism and lowers LDL levels. We observed all these effects in the changes of the patients’ plasma lipid profiles. As is known, the increased consumption of n-6 in comparison to n-3 facilitates the occurrence of cancers, mainly in the large intestine, breast and prostate [22]. Gamma-linolenic acid (GLA, C:18:3n-6) is the only one of the n-6 family of fatty acids to inhibit carcinogenesis [23]. After our three-month therapy, we observed increased levels of average chain length fatty acids, but the use of short-chain fatty acids for elongation to long-chain fatty acids was still visible, especially in the concentration of lauric acid (C:10:0) and heptadecanoic acid (C:17:0). The concentration of stearic acid (C:18:0) increased to the level registered for the control group, which could suggest that the synthesis of oleic acid (C:18: 1w9) derivatives, such as eicosanoic acid (C:20:1 cis11) and eicosadienoic acid (C:20: cis11), had been silenced.

The synthesis of gamma-linolenic acid (GLA, C:18:3n-6) significantly increased, although its concentration before the diet was already high. GLA was formed through desaturation from linolenic acid (C:18:2n6). This can be justified by the fact that gamma-linolenic acid (GLA, C:18:3n-6) is not present in rapeseed oil, and thus was not delivered with the diet [24]. It could be that alpha-linolenic acid n-3 (C:18:3n-3), delivered to the diet with rapeseed oil, did not enhance the synthesis pathway for EPA (C:20:5n3), which is suggested by its low % content in all fatty acids. However, it could, in fact, have been elongated to DHA (C:22:6w3), since an increased level was also observed. To summarize, the diet, through enhancing synthesis pathways for anti-inflammatory factors – the derivatives of gamma-linolenic acid (C:18:3n6), eicosanoic acid (C:20:1 cis11), oleic acid (C:18:1w9), and arachidonic acid (C:20:4n6) – could lead to the inhibition of the synthesis of nervonic acid (C:24:1). It could also contribute to the stabilization of my elinisation, which leads to the decreased level of nervonic acid [25]. The significant increase in the synthesis of arachidonic acid enabled the synthesis of prostaglandins (PG), 2-series thromboxanes (TX), 4-series leukotrienes (LT), and 12 and 15 HETE [26]. The mechanisms of these reactions are based on the activity of cyclooxygenase (COX), with the formation of PG and TX, and lipoxygenase catalysing the synthesis of noncyclic compounds: LT, HPETE and HETE. To summarize, we cannot unambiguously justify the drastic decrease in the level of nervonic acid because its source in PCOS has not yet been described in the literature, and the number of studies related to modifying the fatty acid profile with diet is limited.

### 4.2 Comparison of blood biochemical parameters before and after dietary intervention

Improvement in anthropometric parameters/biometrical differences of women with PCOS has also been observed by other studies [15-16]. This result is even more beneficial because it improves the self-esteem of the patients, increases their self-approval and thus motivates them for further work, decreasing the chances of depression to which patients with PCOS are susceptible [27]. The reduction diet, even though it was based on a low glycaemic index, did not significantly reduce the parameters of carbohydrate metabolism (glucose and insulin measured in fasting state). Numerous authors also studied the influence of starvation diets and fasting on metabolic functions [28]. Various forms of fast did not have an influence on the improvement of the parameters of glucose homeostasis and lipid profile. However, antioxidant improvement was observed, measured by the level of glutathione and nitric oxide and through the suppression of the inflammatory reaction by reducing hs-CRP. The total antioxidant capability did not change [29]. Moreover, it did not lead to a significant decrease of the level of HDL, which shows that not all dietary recommendations were used. Because the concentrations of LDL and TG were dramatically decreased, we think that the patients reduced their consumption of animal products and simple sugars and consumed the recommended vegetable oils, but did not increase their physical activity, despite previous claims. It is known that physical activity contributes to the increase of HDL and the improvement of insulin resistance [30]. To enhance the effects of the diet, patients should be more encouraged to undertake various types of physical activity because the current recommendations were insufficiently motivating. Perhaps training in groups would be necessary.

The lack of significant changes in the androgen hormonal profile is, according to us, caused by its high stability in comparison to the lipid profile. Therefore, dietary treatment of women with PCOS should last longer than three months. No significant differences in hormonal parameters were observed by other researchers using a high-protein diet for one month [31]. However, after three months and using a diet substituted with PUFA, Kasim-Karakas et al. observed a reduction in androgens [7]. We still think that this diet is the best therapy for women with PCOS, especially for those with the proper level of androgens and increased fatty tissue composition.

Because caloric restriction and physical activity, without changes in the ratio of macronutrients, provide observable results in body composition, as seen in anthropometric and biochemical parameters, the most appropriate means of nutrition is a rational diet reducing the content of high-GI carbohydrates and leading to the formation of proper dietary habits [31]. Recommending unconventional diet therapies to women with PCOS, who are exposed to the occurrence of metabolic syndrome and its consequences, can be additionally harmful to an already unbalanced metabolism. Considering the balanced levels of saturated and monounsaturated fatty acids and the lower ratio of PUFA, we must admit that some of the patients probably did not adhere to all recommendations and did not supplement EPA and DHA in the form of cod-liver oil [32]. According to us, this is the main reason for the low level of these fatty acids and the lack of significant reduction of androgens observed by other authors in diets used for the same period of time [7]. At the same time, to prove the effect, it would be advisable to increase the group of examined women using all of the dietary recommendations.

**Authorship**: Research idea and study design: MS, MZCh; literature search: MS; data analysis and interpretation: MS, AD; evaluation of study quality and bias: MS, AD, MZCh, DM, AS, ES; supervision and mentorship: MS, ES. Each author contributed important intellectual content during the manuscript drafting and revision and accepts accountability for the overall work by ensuring that questions pertaining to the accuracy and integrity of any portion of the work are appropriately investigated and resolved.

## Abbreviations

LDL-: low-density lipoprotein
TG-: triglycerides
HDL-: high-density lipoprotein
WC-: Waist Circumference
HC-: hip circumference
FM-: fat mass
BMI-: body mass index
WHR-: Waist-hip ratio
SFA-: Saturated fatty acid
MUFA-: monounsaturated fatty
PUFA-: polyunsaturated fatty acids
UFA-: Unsaturated fatty acid
GI-: glyceamic index
PCOS-: polycystic ovary syndrome
EPA-: eicosapentaenoic acid
DHA-: docosahexaenoic acid
ECLIA-: electro-chemiluminescence immunoassay
ELISA-: enzyme-linked immunosorbent assai
GC-: Gas chromatography
